# A case of intraductal papillary-mucinous neoplasm of the pancreas penetrating into the stomach and spleen successfully treated by total pancreatectomy

**DOI:** 10.1186/s40792-018-0525-1

**Published:** 2018-09-15

**Authors:** Takashi Harino, Yoshito Tomimaru, Kozo Noguchi, Hirotsugu Nagase, Takayuki Ogino, Masashi Hirota, Kazuteru Oshima, Tsukasa Tanida, Shingo Noura, Hiroshi Imamura, Takashi Iwazawa, Kenzo Akagi, Masashi Yamamoto, Tsutomu Nishida, Hiromi Tamura, Shiro Adachi, Keizo Dono

**Affiliations:** 1 Department of Surgery, Toyonaka Municipal Hospital, 4-14-1 Shibahara, Toyonaka, Osaka, 560-8565 Japan; 2 Department of Gastroenterology, Toyonaka Municipal Hospital, Toyonaka, Japan; 3 Department of Pathology, Toyonaka Municipal Hospital, Toyonaka, Japan

**Keywords:** Intraductal papillary-mucinous neoplasm, Pancreas, Penetration, Fistula

## Abstract

**Background:**

Intraductal papillary-mucinous neoplasms (IPMNs) are potentially malignant intraductal epithelial neoplasms that sometimes penetrate into other organs. To the best of our knowledge, no report has yet described a case with penetration into the spleen. We recently encountered a case of IPMN with penetration of the stomach and spleen that was successfully treated by total pancreatectomy.

**Case presentation:**

A 70-year-old female visited our hospital with a complaint of fever and abdominal pain. Contrast-enhanced computed tomography (CT) revealed dilatation of the main pancreatic duct in the entire pancreas and penetration into the stomach and spleen. Upper gastrointestinal endoscopy revealed mucin extruding from four openings of the fistula in the stomach. No malignancy was detected based on cytology of the mucin. Inflammation markers and tumor markers (CEA, CA19–9) were elevated in the blood. The pre-operative diagnosis was IPMN of main pancreatic duct type penetrating into the stomach and spleen. A total pancreatectomy and splenectomy were performed, combined with distal gastrectomy including resection of the fistulas between the pancreas and stomach. No postoperative complications were noted. Histopathological examination of the resected specimen revealed atrophy of the pancreatic parenchyma, and the main duct of the pancreas was filled with mucin. Mucin-producing malignant tumor cells were detected in the epithelium of the main pancreatic duct with no signs of invasion. No malignancy was found at the fistulas between the pancreas and stomach or spleen. The patient was finally diagnosed with non-invasive intraductal papillary-mucinous carcinoma (IPMC) of main pancreatic duct type. Mechanical penetration was suspected as a mechanism of the penetration. The patient remained disease-free without evidence of recurrence more than 15 months after the operation.

**Conclusion:**

Though IPMNs sometimes penetrate into other adjacent organs, penetration into two organs, including the spleen, is rare. The rare case of IPMC penetrating into the stomach and spleen presented here was treated successfully by total pancreatectomy.

## Background

In 1982, intraductal papillary-mucinous neoplasm (IPMN) was reported by Ohashi et al. as a mucus-producing pancreatic carcinoma characterized by a favorable prognosis [1]. IPMNs are potentially malignant, grossly visible intraductal epithelial neoplasms composed of mucin-producing columnar cells. The lesions exhibit papillary proliferation, cyst formation, and varying degrees of cellular atypia. IPMNs can be classified into three types based on imaging studies and/or histopathology: main duct, branch duct, and mixed type.

Kimura et al. initially reported nine cases of IPMN penetrating into other organs, such as the common bile duct, or developed fistula formation [2]. Kobayashi et al. reported that the incidence of fistula formation is 6.6% (18 of 274 cases) [3]; the organs penetrated were the duodenum (67%), stomach (44%), common bile duct (33%), colon (6%), and small intestine (6%). Notably, 39% of the cases with fistula formation developed into multiple organ fistula formation. To the best of our knowledge, there have been no reports of penetration into the spleen. In this context, penetration into multiple organs including the spleen is very rare. Here, we report a case of IPMN with penetration not only into the stomach, but also the spleen, that was successfully treated by total pancreatectomy.

## Case presentation

**Fig. 1:**
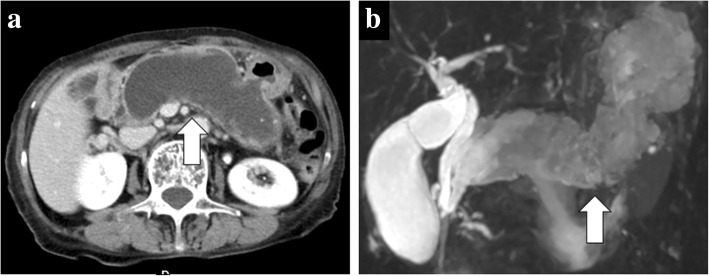
Dilatation of the main pancreatic duct in the entire pancreas. **a** Contrast-enhanced computed tomography revealed a markedly dilated main pancreatic duct (55 mm) and thinning pancreatic tissue (white arrow). **b** Magnetic resonance cholangiography revealed main pancreatic duct dilatation (white arrow)

**Fig. 2:**
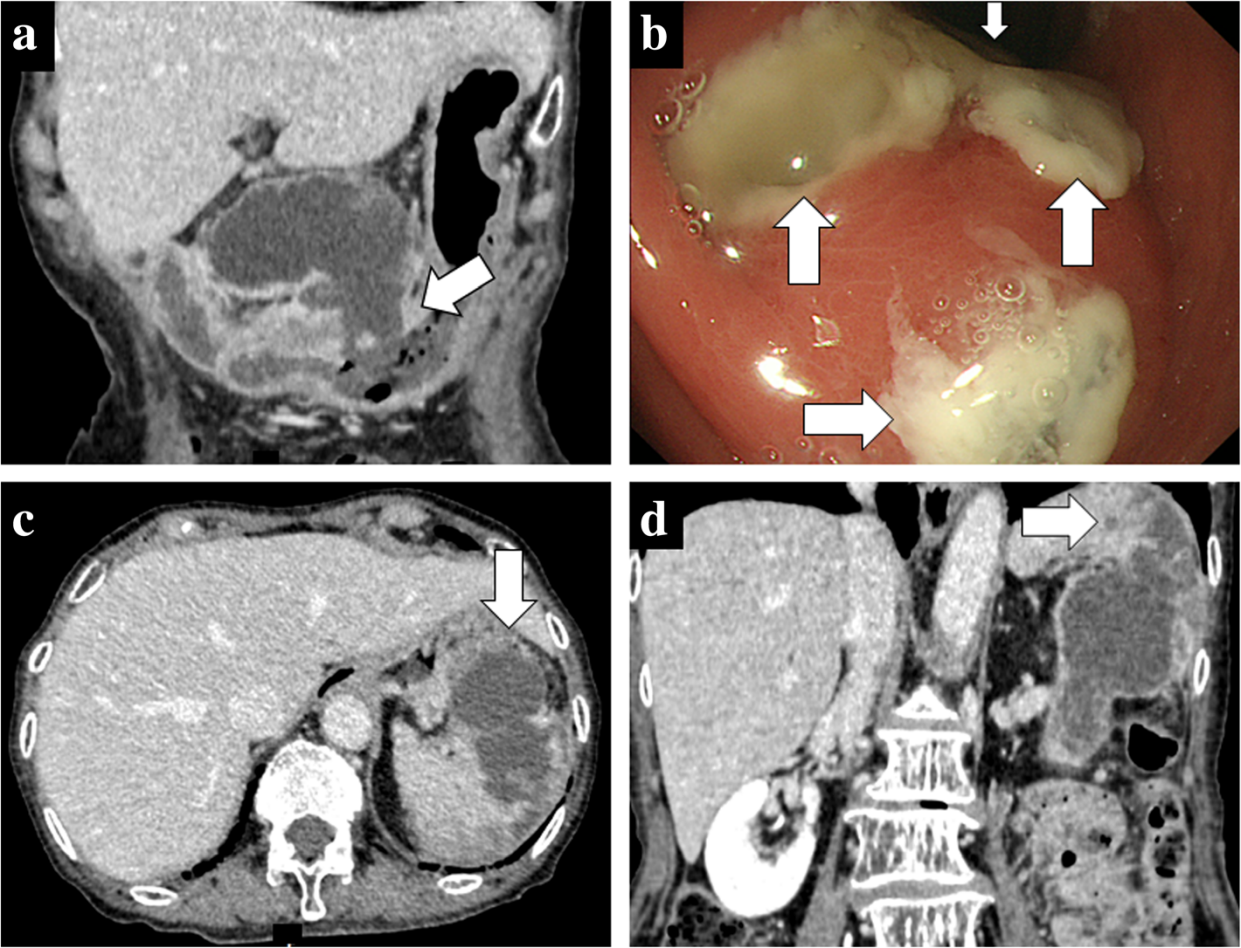
Gastropancreatic fistula and splenopancreatic fistula. **a** Contrast-enhanced computed tomography (CT) revealed fistulas (white arrow) between the pancreas and stomach. **b** Upper gastrointestinal endoscopy revealed four gastropancreatic fistulas on the posterior wall of the gastric body (four white arrows) and mucus discharge from the gastropancreatic fistulas. **c**, **d** Contrast-enhanced CT revealed fistulas (white arrows) between the pancreas and spleen

**Fig. 3:**
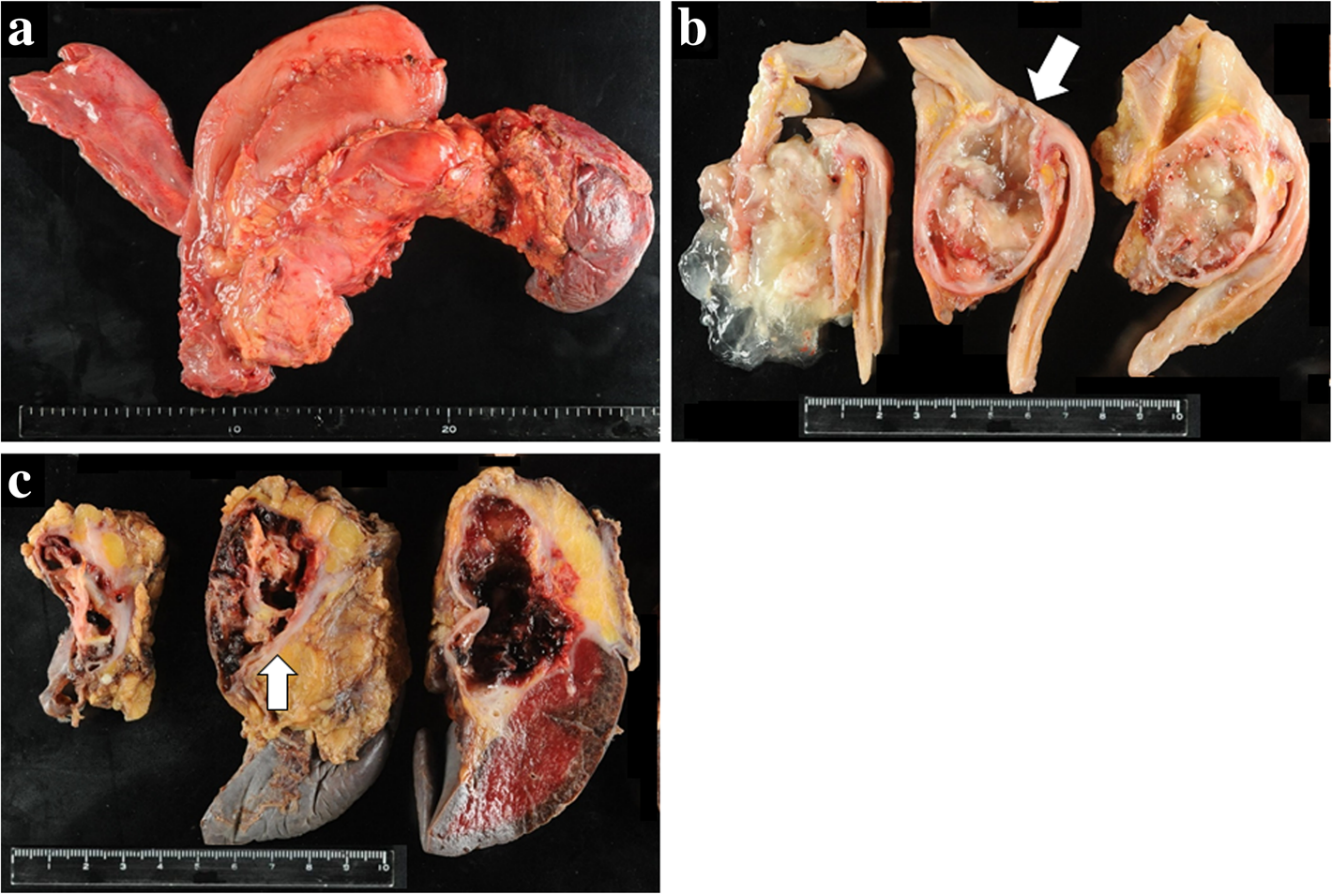
Macroscopic findings for the resected specimen. **a** Most of the cut surface of the whole pancreas was occupied by the dilated main pancreatic duct and the mucus accompanied by atrophy of the pancreatic parenchyma. **b** A gastropancreatic fistula was identified between the main pancreatic duct and the posterior wall of the gastric body. **c** A splenopancreatic fistula was identified (white arrow), and bleeding and infarction were detected in the spleen with mucus penetration

**Fig. 4:**
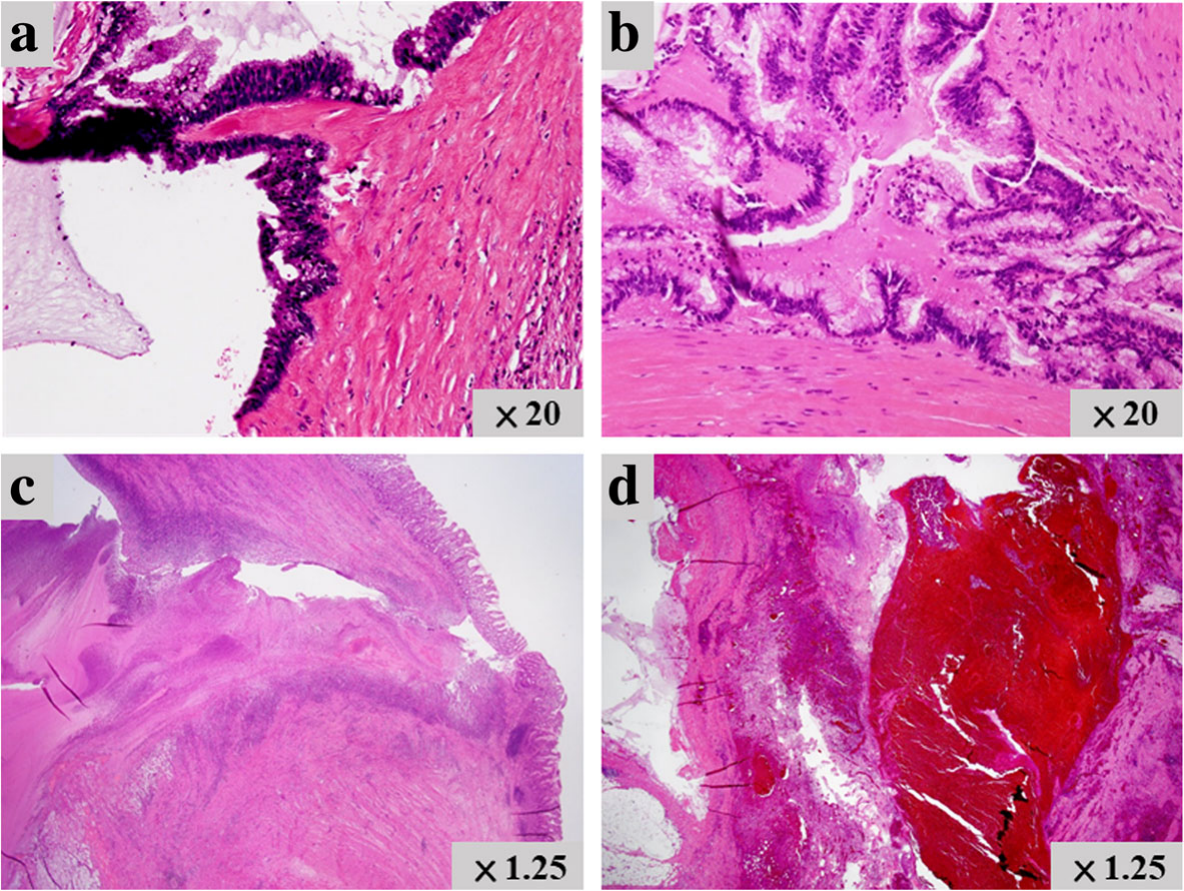
Microscopic findings of the resected specimen. **a** Cancer cells were detected in the epithelium of the main pancreatic duct, though there were no signs of invasion. **b** Adenoma was identified on the remaining part of the epithelial cells of the pancreatic duct. **c** A gastropancreatic fistula was microscopically detected, and no cancer cells were detected on the area. **d** Microscopic examination identified a splenopancreatic fistula where no cancer cells were detected

A 70-year-old woman was admitted to our hospital because of upper abdominal pain. Her medical history included appendicitis at 20 years old. Upon physical examination, left hypochondriac pain and tenderness in the upper abdomen were noted. The laboratory examinations revealed elevated inflammatory markers (white blood cell count = 13400/μL, C-reactive protein = 11.58 mg/dL) and biliary enzymes (lactate dehydrogenase = 250 U/L, alkaline phosphatase = 535 U/L, γ-glutamyltranspeptidase = 76 U/L). The levels of tumor markers were also elevated (carcinoembryonic antigen = 9.4 U/mL, cancer antigen 19-9 = 550 U/mL). Pancreatic tumor markers were not elevated (s-pancreas-1 antigen = 20.0 U/mL, duke pancreatic monoclonal antigen type 2 ≤ 25 U/mL). Contrast-enhanced computed tomography (CT) revealed a markedly dilated main pancreatic duct (MPD) 55 mm in length in the whole pancreas, and the whole pancreatic parenchyma was thinning with atrophy (Fig. 1a). In addition, gastropancreatic fistula and splenopancreatic fistula were detected, suggesting penetration of the pancreatic tumor (Fig. 2a, c, d). As seen on the CT examination, dilatation of the MPD was detected on magnetic resonance imaging, and its content was visualized using low signal intensity on T1-weighted images and high signal intensity at T2-weighted images (Fig. 1b). The wall of the MPD and fistula had high signal intensity on diffusion-weighted images. Upon examination by upper gastrointestinal endoscopy, four gastropancreatic fistulas were identified on the posterior wall of the gastric body and mucus discharged from the gastropancreatic fistulas (Fig. 2b). Cytological examination of the mucus did not reveal any signs of malignancy. On the basis of the findings, the patient was pre-operatively diagnosed with IPMN of main ductal type penetrating into the stomach and spleen and surgery planned for her treatment. A total pancreatectomy, splenectomy, and distal gastrectomy combined with resection of the fistulas were performed. Considering the malignant potential based on the main ductal type with > 10 mm MPD dilatation, we also performed lymphadenectomy. The total operation time was 426 min, and the total intraoperative blood loss was 575 mL. Macroscopic examination of the resected specimen indicated swelling of the whole pancreas. When the resected specimen was divided, mucus swelled out, and then most of the cut surface of the whole pancreas was occupied by the dilated MPD and the mucus accompanied by atrophy of the pancreatic parenchyma (Fig. 3a). The gastropancreatic fistula (Fig. 3b) and splenopancreatic fistula (Fig. 3c) were macroscopically identified. In the spleen, bleeding and infarction were detected in addition to the mucus penetration. Microscopic examination of the resected specimen revealed cancer cells in the epithelium of the MPD in part of the tumor (Fig. 4a). There was no sign of infiltration on the cancer cells, and the remaining part of the MPD epithelium was adenoma (Fig. 4b). At the gastropancreatic fistula (Fig. 4c) and splenopancreatic fistula (Fig. 4d), no cancer cells were detected, only mucus and inflammatory cells. Finally, we diagnosed the tumor as non-invasive intraductal papillary-mucinous cancer (IPMC) of the pancreas. No postoperative complications were noted. The patient has remained disease-free without evidence of recurrence for 15 months.

### Discussion

During the last three decades, an increasing number of reports of IPMN of the pancreas have been published [2, 4, 5]. Though IPMNs originate from the pancreatic duct cells similar to invasive ductal adenocarcinoma of the pancreas, IPMN exhibits a unique clinical feature different from invasive ductal adenocarcinoma, such as secretion of a large quantity of mucin by the neoplasm, and slow and expansive growth associated with low malignant potentials for metastasis and invasion compared to invasive ductal adenocarcinoma. Fistula formation into other organs is also one of the characteristic features of IPMNs. With regard to its incidence, Kimura et al. initially reported nine cases with IPMN which penetrated into other organs such as common bile duct or developed fistula formation [2]. Kobayashi et al. also investigated that the incidence of the fistula formation was 6.6% (18 out of 274 cases) [3]. The organs penetrated were also reported in their investigation: duodenum (67%), stomach (44%), common bile duct (33%), colon (6%), and small intestine (6%). Notably, 39% of the cases with fistula formation developed into multiple organs fistula formation. In the report, the spleen is not reported as the organ penetrated into by IPMN, and furthermore, to the best of our knowledge, there have been no reports describing IPMN cases penetrating into the spleen. In this context, our IPMN case, which exhibited penetration into multiple organs including the spleen, is very rare, suggesting significance of reporting the case. The pathogenesis of fistula formation in IPMN is generally considered to be divided into two main types based on the underlying mechanism: invasive penetration of cancer cells and mechanical penetration. Though invasive penetration is derived from direct invasion of organs by cancer cells, mechanical penetration is due to the high inner pressure of a mucus-filled pancreatic duct [3, 6]. Kobayashi et al. reported that three out of nine cases (33%) had invasive penetration, and mechanical penetration was shown in the remaining six cases (67%) [3]. In the current case, cancer cells did not exist in the area of the fistulas, suggesting mechanical penetration as the underlying mechanism in the development of the fistula. Our finding that the mucus in the MPD swelled out when the resected specimen was divided may be associated with the high inner pressures, which may support mechanical penetration as the pathogenesis of fistula formation in this case. Several previous studies have reported that inflammation is also involved in mechanical penetration [7–10]. Based on our finding of inflammatory cells at the fistulas in this case, inflammation may exist at the fistula, resulting in mechanical penetration. Furthermore, when considering mechanical penetration apart from invasive penetration, a pressure gradient seems to be necessary for fistula development. Lumen organs, including the duodenum, stomach, and bile duct, may easily be under lower pressure than solid organs, such as the spleen, which could be one reason why fistula formation into the spleen is rare compared to the lumen organs. Kawarada et al. reported a 5-year survival rate of IPMC with penetration of 46.5% in Japan [11]. Kimura et al. reported a 5-year survival rate of IPMC with penetration or invasion of neighboring organs of 28% [2]. In our case, the follow-up period was just 15 months. Although the previously reported prognosis might not be applied to our case since the abovementioned prognosis was concerning about cases with invasive carcinoma, not about non-invasive carcinoma, further observation would be necessary in our case.

## Conclusions

We experienced a case of IPMN of the pancreas penetrating into the stomach and spleen that was successfully treated by total pancreatectomy. This case could contribute to improving our understanding of this type of neoplasm.
